# Observations on a Case of Catalepsy

**Published:** 1812-11

**Authors:** 


### *To the Editors of the Medical and Physical Journal.*

"Quand la foiblesse des hommes n'a pu trouver les veritables causes, leur subtilitè en a substituè d'imaginaries, qu'ils on exprimeès par de noms specieux qui remplissent les oreilles, et non pas l'esprit."

GENTLEMEN,

AN attempt, in your last Journal, by Dr. Wilmer, of Coventry, to support Dr. Balfour's hypothesis concerning sol-lunar influence, by detailing a case of Catalepsy which occurred to him some years ago, has attracted my notice, less by its singularity, than the singularity of the treatment, and medical opinions, there given. I must premise the observations I have ventured to make, by declaring, that the parties are unknown to me; that I am actuated by no motive but the advancement of professional knowledge; and, that it is alone the respectable rank which Dr. Wilmer holds holds as a physician, that induces me to question practice and opinions which might be a sanction to others.

I hold it to be of no practical consequence, whether the disease made its attack at the full moon, at the end of every week, of every day, or of every hour. Nor it is of moment to know whether the toes were turned out or in, whether the eyes were open or shut; for the varieties of contortions in convulsive diseases are endless. The cause is first to be investigated. It is a fact well known, that convulsions in children, when not produced by organic disease, or teething, neither of which were the case here, are generally owing to a disordered state of the alimentary canal, from whatever causes produced; though the most common are bad air, improper diet, &c. which, by checking some secretions, and altering the nature of others, cause numberless modifications of convulsive disease. In this instance, the child had been weaned between the third and fourth month, thereby increasing the chance of disordered stomach and bowels, to which I attribute the disease; and of which the flatulence and distension, which preceded a fit, were excellent indications.

Let us next examine the treatment. A Galbanum plaster was applied to the stomach, which could do no good, and, to say the least of it, must be superfluous and unpleasant. Two grains of calomel, with two of musk, were next ordered, and a cathartic draught to follow. A bath, prepared from a decoction of rosemary, was also advised. Evacuations by stool being so strongly pointed out as the only treatment likely to be useful, and for that purpose the simplest mode the best, one sees with astonishment two grains of musk ordered along with the calomel, the use of which, unless to render the other unpalatable, does not appear. I am ignorant too, that a decoction of rosemary has any preference, as a bath, over simple water, which is much easier procured. The report three days afterwards points out, in the clearest manner, the source of the disorder, where it mentions "many stools having been evacuated, *some of which were clay-colored,*" shewing the functions of the liver to be diseased. The very judicious treatment of giving calomel with rhubarb every second morning, was then adopted; the consequence of which was, that, after two doses had been given, a convulsion on the 12th lasted only five minutes, whereas they used to continue forty minutes. Now, in place of prosecuting a plan of treatment which had already produced so considerable a reduction in the duration of the fit, the apothecary orders a blister to the back, and a mixture with valerian and assafœtida internally, the former to give unnecessary tor-

 ture, ture, and the latter sickness and disgust. On the following day the fit returned, shewing no respect to, or rather in consequence of, the blister and abominable mixture, but only lasted, like the former, for five or six minutes. Again Dr. Wilmer prescribes two grains of calomel, with two of musk, to be taken every second day under the form of *pill*—the child five months old! Besides, a mixture composed of castor oil, musk mixture, and fœtid spirits of ammonia, in which the castor oil, the only medicine useful, except to create nausea, sickness, and disorder, was to be taken in doses under *eight drops*, every fourth hour. A glyster of assafœtida was also ordered to be given every morning; with what intention it is impossible to guess, for it could not be to have its action as a purgative, which in that way it is, though apparently unknown to the prescriber; otherwise surely a less unpleasant one might, if at all necessary, have been substituted. Three days now elapsed without the recurrence of any fit, when the apothecary writes to Dr. Wilmer that "the fœtid glysters appear to be of great service: they always bring off a quantity of wind, and a copious stool, which appears to consist of much coagulated milk. *The first brought away two stools colored with bile.* I have not repeated the calomel, as Mr. and Mrs. H. are afraid of its effects."

It is very clear that the calomel already given had removed the complaint, which the appearance of the bile plainly testified. It is not probable the coagulated milk had lodged in the intestinal canal; but is it possible that the child was allowed to continue a milk diet under such a disease, and especially such medicines? This is, however, still exceeded by Dr. Wilmer's reply to Mr. Bucknell. "I do not wish (he says) to press the farther employment of calomel, nor can it be much longer continued without danger of affecting the mouth! I am of opinion also, that now we can only expect advantage from it as an *evacuant*, a service which may be derived with more *safety* from other quarters." Again, "*I have suspected coagulated milk, and the immediate cause of the disease appears to have arisen from an accumulation of acid acrimony in the stomach and intestines, acting upon a nervous system under the influence of morbid idiosyncracy.* I wish to continue the Mosch, under the following form:

℞. Sap. dur. ℈ij. Mel. ʒij. Vitel. ovi. Sodæ ppt. ʒſs.

Mist. Mosch. ℥ij. M. Sumat ℥ſs. ter quotidie."

I have copied the foregoing as a specimen of pharmaceutical composition, leaving your readers to judge how far it combines simplicity, elegance, and utility. Let us mark its effects.

"20th.

"20th. During the last four days spasms have frequently returned. She is still costive." To remove this a blister was applied to the neck, and the following mixture prescribed:

℞. Potass. Subcarbon. gr. xv.

Syr. Rosæ ℥fs.

Tinct. Jalap. ʒifs.

Mist. Mosch. ℥ij. M. ℥fs. ter die.

"Excoriation now prevented the continuance of the glysters."

It is plain the tincture of jalap was the only remedy that could be useful; yet what a novel mode of removing costiveness in an infant, and in the proportion *of eighteen drops* three times a day!

"26th. Better in all respects; spasms less frequent and violent.

"30th. In two days more the period will arrive when the grand (or lunar) attack may be expected. In order, therefore, to prepare for it, and to lessen the morbid irritability of the part where the disease appeared to originate, and of the system in general, I directed as follows:

℞. Assafœtid. ℈ij.

Tinc. Opii gtt. x.

Muc. Arab. ℥fs.

Aq. Pur. ℥ij. Mft. enema horâ 7ma matutina injeciend.

Repetatur ad quatuor vices.

℞. Opii Pur.

Camphoræ aa ʒi.

Theriac. Androm. ℥fs. Mft. pasta applicetur scrobiculo cordis."

However, the disease having been already removed by the calomel, no grand attack came on, and a return to good health was the consequence.

Permit me now to ask Dr. Wilmer, whether he ever saw or ever heard of any child, whose mouth was affected by calomel given in any quantity, at that age, or even years afterwards?—certainly never; and of late it has been fully tried in hydrocephalus and croup. The fear was, therefore, a vain one; by which means, the medicine which had then, but for improper interference, effected a cure, was deserted for blind and groping chance. Nor do I comprehend what other advantage had been gained, or expected from it, than as an *evacuant*. I would also ask Dr. Wilmer what he means by having "suspected coagulated milk," and whether he thought assafœtida, musk, &c. likely to remove this? To be sure, suspecting coagulated milk after being told of *its*

*appearance*, reminds me of the sapient opinion that the lamb was suspected of being dead when its skin was found! Nor do the mysticisms of Galen or Paracelsus contain a darker sentence than the following, which must have given Mr. B. a very accurate idea of the nature of the complaint—"*the immediate cause of the disease appears to have arisen from an accumulation of acid acrimony in the stomach and intestines, acting upon a nervous system under the influence of morbid idiosyncracy.*"

Is it possible that in the nineteenth century medical men, and of eminence too, can write, or even think, such chaotic nonsense! No wonder that medicine, of all the arts, remains unimproved, when such jargon is tolerated for information. Would it not be better that, descending from such *lunary* conjectures, we more diligently observed the simple facts before us, and stated them as they occurred under the *influence* of common sense.

I believe most of your readers will agree with me in thinking this case only a variety of convulsion, proceeding from derangement of the alimentary canal, which required no uncommon treatment nor empty hypothesis to explain. Most men would have given one or two grains of calomel, with or without some purgative adjunct, every night at bedtime; and assisted its action on the bowels by some simple cathartic, such as senna infusion, next day; and continued every day a similar treatment until the stools were natural, when, as naturally, the convulsions would cease. A short continuance would have cured the complaint; and, had Dr. Wilmer, or Mr. Bucknell, at their usual visits, been watching the evacuations for that event, they might afterwards have defied the *moon, however full*, from disturbing their patient's rest, or their own, on her account!

No man, who volunteers to appear before the public, thinks himself deficient in that which he *chooses* to tell. His mind ought to be prepared for the consequences, and he must expect criticism. Should that be candid and just, he has no right to complain; but, within these boundaries, it is necessary that severity should be used, to extirpate the fungus which so luxuriantly envelopes and obscures almost every medical production of the present day. Trusting that I have not exceeded these premises, and that freedom of discussion, and the improvement of medical science, are no less your objects than mine, I have to solicit the insertion of this in your next Number, which shall not be the last contribution of, Gentlemen,

Your most obedient Servant,

*London, Sept.* 12, 1812. *A Practical Physician.*

